# To the editor

**DOI:** 10.1186/s13613-024-01404-0

**Published:** 2024-12-18

**Authors:** F Duprez, S Zacharis, J Roeseler

**Affiliations:** 1 ICU and Research Unit and Innovation Condorcet-Epicura. Epicura Hospital, Hornu, Belgium; 2 Respiratory Medicine Unit. Epicura Hospital, Hornu, Belgium

We read with great interest the paper by Helms et al. on “Oxygen therapy in acute hypoxemic respiratory failure: guidelines from the SRLF-SFMU consensus conference” [1]. In their article, the authors clarify the role of oxygen therapy and its devices in patients with acute hypoxemic respiratory failure (ARF). The authors present 22 statements in response to 11 questions regarding the management of ARF. The article is fascinating and should optimize the use of oxygen during ARF.

However, we have some comments on a concept discussed in this article, particularly on the FiO2 delivered by oxygen masks. The authors argue that the FiO2 of patients treated with standard mask oxygen therapy can be estimated using the Coudroy formula [2]:

$${\text{FiO}}2\left( \% \right) = 21 + \left( {{\text{QO}}2 \times 3} \right)$$

where QO2 is the oxygen flow rate in L/min.

We disagree with this information for several reasons:

Although Coudroy et al. published a clinical study on FiO2 estimation during oxygenation using a non-rebreathing mask, the study did not involve a standard mask as mentioned in the article. Moreover, the results of measuring FiO2 in clinical research by Coudroy et al. showed large variability, which may lead to erroneous interpretations of the true value of FiO2 in clinical settings [2].

FiO2 Prediction Formula: FiO2(%) = 21+(QO2 × 3): This is the Helms article is originally attributed to Professor JL Vincent and is associated with oxygenation via nasal cannula [3].

Contradiction in Helms et al.: In the article by Helms et al., we identified a contradiction between their statement and the following sentence: “The FiO2 delivered depends on the minute ventilation and the seal if a mask is used”. A prediction formula for FiO2 that solely relies on the oxygen flow rate would lack precision, potentially resulting in inaccurate assessments of the actual FiO2.

Maintaining a constant FiO2 with low-flow oxygenation systems: Several studies have emphasized the challenges in achieving a stable FiO2 with these systems [4, 5].

Table N°2: This table appears to indicate that FiO2 is independent of inspiratory flow, which contradicts the preceding text.

We believe it is necessary to challenge certain beliefs or contradictions regarding oxygen administration, as this can lead to misjudgments of our patient’s clinical condition in certain situations.

## Electronic supplementary material

Below is the link to the electronic supplementary material.

Supplementary Material 1
