# Supplementary material for: To the editor
Source: Ann Intensive Care. 2024 Dec 18;14:180. doi: 10.1186/s13613-024-01404-0 (PMC11655718; doi:10.1186/s13613-024-01404-0)
Supplement: Supplementary file 1 — Supplementary Material 1 [file 13613_2024_1404_MOESM1_ESM.docx]

To Professor Teboul JL

Editor in Chief

of Annals of Intensive Care

Hornu, the 4 October 2024

Dear Professor, Teboul

We are pleased to submit the attached manuscript for consideration for publication as **letter to editor** in rapport with following article:

Helms J et al. Oxygen therapy in acute hypoxemic respiratory failure: guidelines from the SRLF-SFMU consensus conference. Ann Intensive Care. 2024 Sep 5;14(1):140. doi: 10.1186/s13613-024-01367-2. PMID: 39235690; PMCID: PMC11377397.

We have no conflicts of interest to declare.

We hope that this letter to the editor will be relevant for publication in the Annals of Intensive Care.

Could you please waive the publication fee for my letter, as it is addressed to the Editor-in-Chief.

Thank you for your consideration of our manuscript. We look forward to hearing from you soon.

Yours Sincerely,

Dr Frédéric Duprez

Head of Research Unit Condorcet-Epicura

ICU Epicura Hornu Belgium

frederic.duprez@epicura.be
